# Relationship between bilirubin and systemic lupus erythematosus: A systematic review and meta‐analysis

**DOI:** 10.1002/iid3.1115

**Published:** 2023-12-22

**Authors:** Yanxia Yu, Qiaoyu Wang, Dongmei Zhang, Weihua Wu, Zheng Jiang

**Affiliations:** ^1^ Department of Nephrology The Affiliated Hospital of Southwest Medical University Luzhou China; ^2^ Sichuan Clinical Research Center for Nephropathy Luzhou China

**Keywords:** lupus nephritis, serum bilirubin, systemic lupus erythematosus

## Abstract

**Aims:**

Systemic lupus erythematosus (SLE) is an autoimmune disease with a high prevalence worldwide. This study aimed to examine the correlation between serum bilirubin levels and SLE.

**Methods:**

The Cochrane library, Embase, PubMed, and China National Knowledge Infrastructure (CNKI) databases were examined and assessed until March 2023. RevMan 5.3 software was utilized for the analysis of clinical trails.

**Results:**

Five case‐control studies were chosen and incorporated, examining the levels of serum bilirubin in patients with SLE compared to healthy individuals, as well as in active SLE patients versus inactive ones, in different sexes and in SLE patients with or without lupus nephritis (LN). The results of this meta‐analysis demonstrated that serum bilirubin in healthy individuals were obviously increased compared to SLE patients (MD = 4.76; 95% CI, 3.15–6.38, *p* < .00001). Additionally, inactive SLE patients had higher levels of bilirubin than active SLE patients (MD = 3.15; 95% CI, 0.46–5.84, *p* = .02), and SLE patients without lupus nephritis had higher levels of serum bilirubin than those with lupus nephritis (MD = 4.91;95% CI, 2.87–6.95, *p* < .00001). Nevertheless, there were no disparities observed among SLE patients of varying sexes (MD = 0.34; 95% CI, −0.01 to 0.69, *p* = .06).

**Conclusion:**

The concentration of serum bilirubin may potentially be used as an indicator for estimating the advancement of SLE and reflecting the presence of kidney complications in individuals with SLE. Furthermore, more high quality studies were needed to identify these findings.

## Graphical abstract

Higher level of bilirubin indicates better outcome of systemic lupus erythematosus.

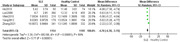

## 1 INTRODUCTION

Systemic lupus erythematosus (SLE) is featured by aberrant activity of the immune system, which is more prevalent in women of childbearing age, leading to renal, neuropsychiatric, dermatological, and cardiovascular damage.^1^, ^2^ The various manifestations present a great challenge to the clinician. Identifying risk factors associated with the advancement of SLE holds significant importance.

The available literature suggests that there is an elevation of oxidative stress in SLE, which leads to dysregulation of immune system, abnormal activation and processing of signals related to cell‐death, and production of autoantibodies.^3^ According to numberous reports, bilirubin is commonly recognized as an antioxidant and immunomodulator, potentially serving as a safeguard aganist autoimmune disease.^4^ It seems related to the disease activity of rheumatoid arthritis, Takayasu arteritis, Crohn's disease, and primary Sjögren's Syndrome.^5^, ^6^, ^7^ Besides, many studies have indicated a strong correlation between SLE and serum bilirubin.^8^, ^9^, ^10^, ^11^, ^12^ However, perhaps due to the sample size and different methodologies, the results of these studies often lack of enough reliability for widely application. Therefore, this study was carried out as a meta‐analysis to synthesize these previous studies for further exploring the relationship between SLE and serum bilirubin.

## 2 METHODS

### 2.1 Search strategy

PubMed, Embase, Cochrane Library, and China National Knowledge Infrastructure (CNKI) databases were utilized for conducting a literature search. Relevant trails were identified by searching PubMed (1966 to April 2023), Embase (1974 to April 2023), Cochrane Renal Group (1999 to April 2023), and CNKI. Systemic lupus erythematosus, lupus, lupus nephritis, LN, SLE, bilirubin, and serum bilirubin were among the search terms utilized. Furthermore, we additionally conducted a manual search through the reference lists of articles to discover additional sources. The findings only included studies that were carried out on individuals and published in English and Chinese languages.

### 2.2 Inclusion criteria and risk of bias

The included studies met the criteria for inclusion, which entailed comparing the serum bilirubin levels in individuals with SLE and healthy subjects, patients with active SLE and those with inactive SLE, SLE patients of varying genders, and SLE patients with or without lupus nephritis. The following exclusion criteria were applied: (1) the participants were children or animals; (2) articles that were reviews, editorials, case reports, letters, or conference abstracts; (3) studies for which full texts were unavailable; (4) the participants with liver disease or some other diseases which could effect the concentration of bilirubin. To assess the potential for bias, the Cochrane network suggested utilizing risk of bias tables.

### 2.3 Extraction and orgnization of data

Yanxia Yu and Qiaoyu Wang, two authors, conducted data extraction separately using standard forms. Whenever there was a discrepancy, Dr Zheng Jiang was consulted. In cases where detailed data could not be extracted, the authors were reached out to via emails. For each study included, we recorded foundamental details like primary author, publication year, research methodology, criteria for inclusion, sample size, essential characteristics of study participants, and bilirubin concentration.

### 2.4 Statistical analysis

Analysis was conducted using Review Manager 5.3 software. Risk ratios (RR) and 95% confidence intervals (CIs) were utilized to present the results of binary outcomes. When different scales were used, standardized mean difference (SMD) was employed instead of Mean difference (MD) for results with continuous scales. When bilirubin concentration was showed as median and interquartile range, it could be estimated by mean ± SD according to the study of Wan.^13^ Heterogeneity was analyzed using *I* ^2^ statistical method. The meta analysis was conducted using random‐effects analysis (*I* ^2^ > 50%) and fixed‐effects analysis (*I* ^2^ < 50%) according to the protocol. The *Z* test was utilized to analyze the overall impact, with statistical significance indicated by *p* < 0.05.

## 3 RESULTS

### 3.1 Analysis of chosen studies and features of trials

**Figure 1:**
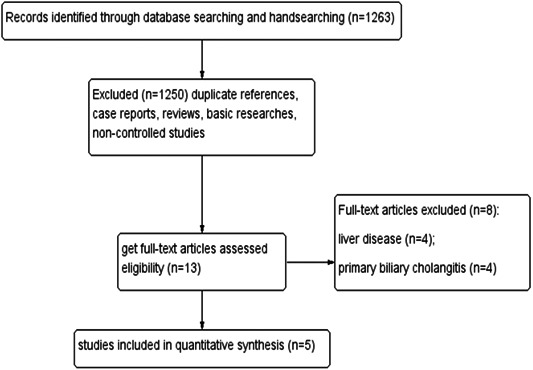
Flow diagram of articles considered for inclusion.

From variable databases, We obtained a total of 1263 articles in the initial search. After a thorough examination of the tittle and abstract, a total of 1250 articles were eliminated due to duplicate references, case reports, reviews, basic research, and noncontrolled studies. Further selection involved identifying the complete texts of the remaining 13 articles. After performing a thorough review, additional eight articles were excluded due to their relevance to patients with liver disease or primary biliary cholangitis as this could potentially effect the bilirubin level based on the strict criteria. In the end, this meta‐analysis and systematic review incorporated a total of five studies. The search approach employed in this study was explained in Figure 1. Table 1 displayed the attributes of the studies that were included. The risk of bias assessment was conducted with the risk of bias table recommended by Cochrane in Table 2.

**Table 1:** Characteristics of studies included.

References	Country	Type	Patients with SLE							Healthy controls		
			Number	Sex (M/F)	Age	SLEDAI	With activity	SLE diagnostic criteria	With LN(%)	Number	Age	Sex (M/F)
He et al.^8^	China	C	50	12/38	35.06 ± 14.36	NR	58%	ACR	100	74	37.99 ± 1.73	19/55
Luo^9^	China	C	366	38/328	31.4 ± 11.5	NR	64.2%	ACR	NR	455	32.2 ± 10.6	45/410
Vitek et al.^10^	Czech	C	218	32/186	39.4 ± 14	NR	NR	ARA	43	180	39 ± 10	87/93
Yang et la.^11^	China	C	179	22/157	36 (24–47)	NR	NR	ACR	55.3	154	38.5 (33–44)	17/137
Zhang et al.^12^	China	C	341	NR	40.46 ± 12.92	8 (3–12)	41.1%	ACR	NR	332	49.10 ± 12.07	NR

Abbreviations: ACR, American College of Rheumatology classification criteria; ARA, American Rheumatism Association criteria; C, case–control study; LN, lupus nephritis; NR, not reported; SLE, systemic lupus erythematosus; SLEDAI, Systemic Lupus Erythematosus Disease Activity Index.

**Table 2:** Risk of bias summary.

References	Random sequence generation	Allocation concealment	Blinding of participants and personnel	Blinding of outcome assessment	Selective reporting	Incomplete outcome data	Other
He et al.^8^	‐	+	‐	‐	+	+	+
Luo^9^	‐	+	‐	‐	+	+	+
Vitek et al.^10^	‐	+	‐	‐	+	+	+
Yang et al.^11^	‐	+	‐	‐	+	+	+
Zhang et al.^12^	‐	+	‐	‐	+	+	+

### 3.2 SLE patients exhibited a reduction in serum bilirubin levels

**Figure 2:**
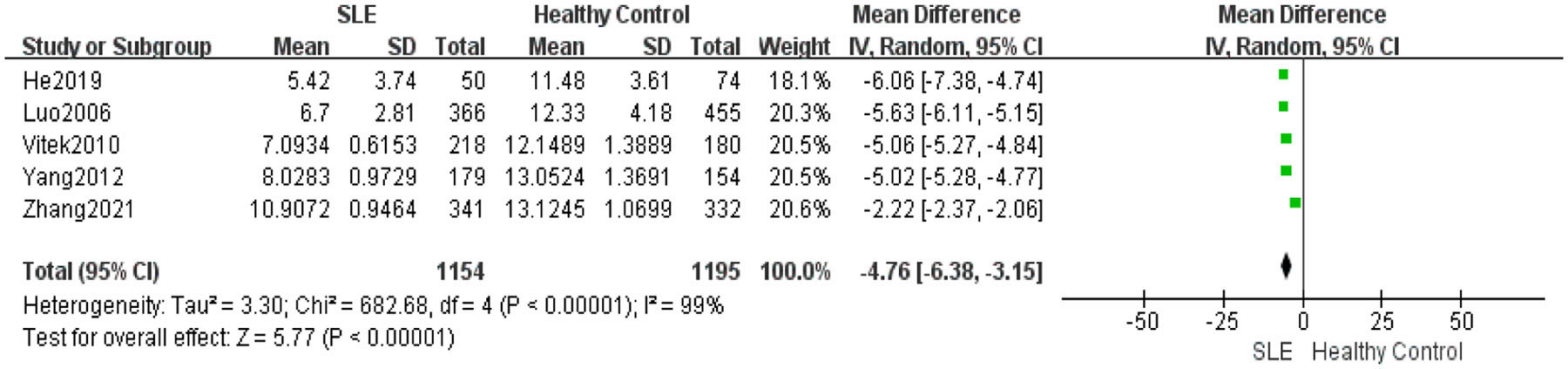
Systemic lupus erythematosus patients exhibited a significant decrease in serum bilirubin levels compared to healthy controls.

To examine the association between serum bilirubin and SLE, we analyzed the disparity of serum bilirubin level among SLE patients and individuals without the condition. This analysis encompassed five case–control studies. The meta‐analysis employed a random‐effects model because the heterogeneity tests indicated *I* ^2^ = 99%. The findings indicated that SLE patients had a considerably reduced serum bilirubin level compared to the control group of healthy individuals (MD = 4.76; 95% CI, 3.15–6.38, *p* < .00001) (Figure 2).

### 3.3 Exacerbation of SLE showed a negative correlation with serum bilirubin levels

**Figure 3:**
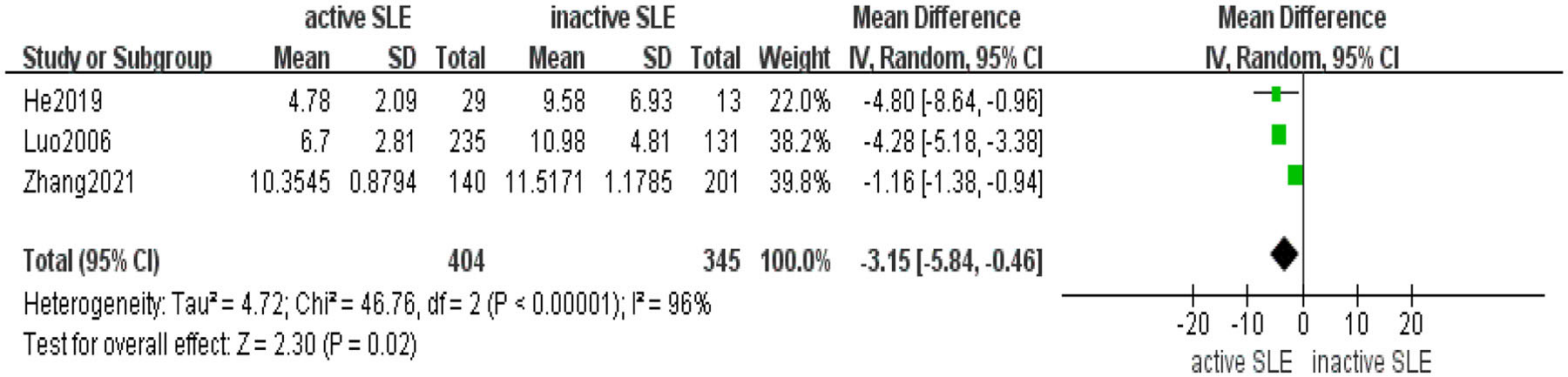
Serum bilirubin levels in active systemic lupus erythematosus patients showed a significant reduction in comparison to levels observed in patients with inactive disease.

The comparison of bilirubin level between patients with active SLE and patients with inactive SLE included three case–control studies. Meta analysis using random‐effects models (*I* ^2^ = 96%) revealed a correlation between active SLE and a decreased level of serum bilirubin (MD = 3.15; 95% CI, 0.46–5.84, *p* = .02) (Figure 3).

### 3.4 Serum bilirubin was related to renal damage of SLE

**Figure 4:**
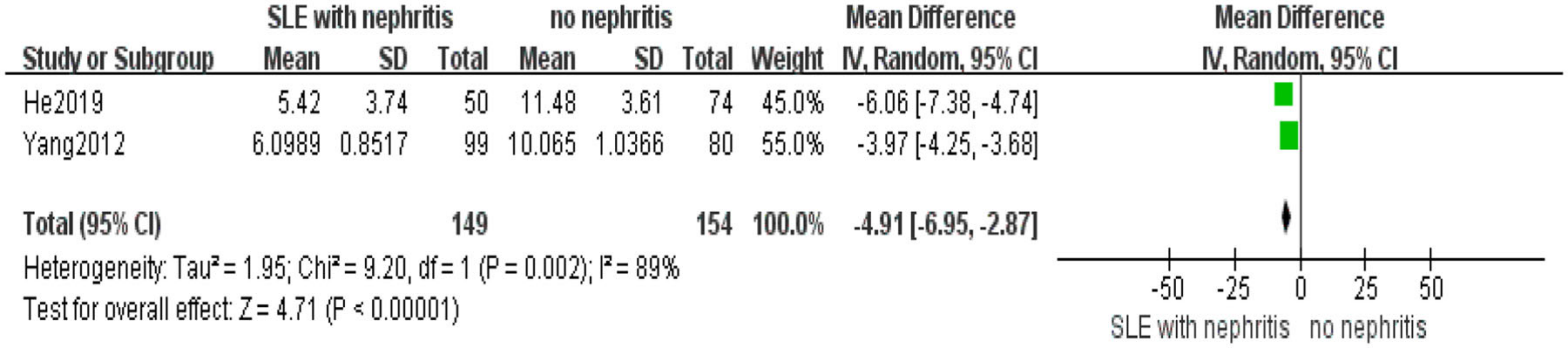
Systemic lupus erythematosus patients with nephritis exhibited a notable decrease in serum bilirubin levels compared to those without nephritis.

Kidney was one of the most common target‐organ druring the development of SLE. Two studies were included to elucidate the impact of serum bilirubin on individuals with SLE and nephritis. A significant difference in bilirubin levels was found between SLE patients with and without nephritis, as determined by a meta‐analysis using a random‐effect model (*I* ^2^ = 89%). It seemed that serum bilirubin may alleviate the renal damage of SLE patients (MD = 4.91; 95% CI, 2.87–6.95, *p* < .00001) (Figure 4).

### 3.5 The correlation between serum bilirubin levels and variations in gender among individuals with SLE

**Figure 5:**
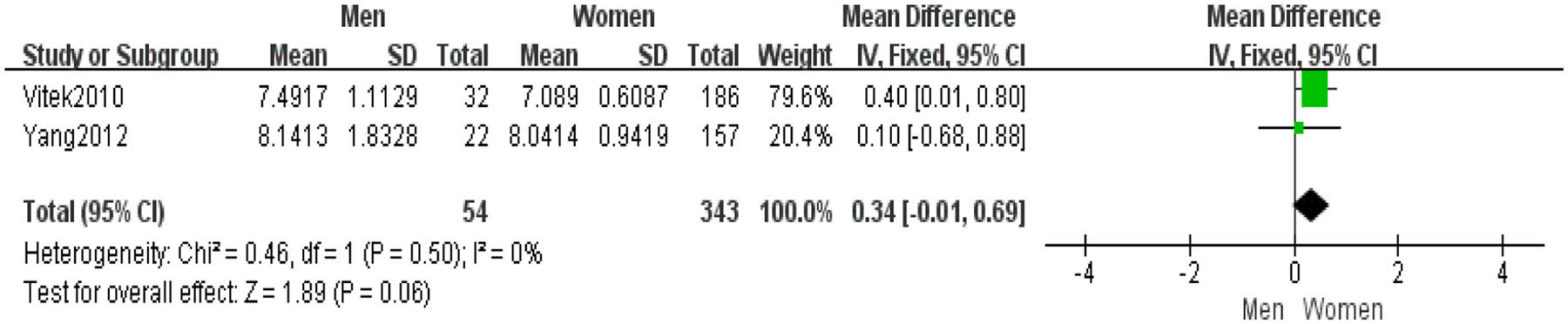
No difference in serum bilirubin between female systemic lupus erythematosus (SLE) patients and male SLE patients. MD, mean difference.

Additionally, we assessed the influence of serum bilirubin in SLE individuals with different genders. A fixed‐effect model was chosen in this meta‐analysis including two case–control studies (*I* ^2^ = 0%). It suggested no difference in bilirubin between female SLE patients and male SLE patients (MD = 0.34; 95% CI, −0.01 to 0.69, *p* = .06) (Figure 5).

## 4 DISCUSSION

SLE is a highly intricate autoimmune disorder characterized by diverse clinical presentations, resulting in dysfunction of various organs such as the dermis, renal system, musculoskeletal system, and nervous system. The disease course can be chronic or characterized by relapses and remissions. Physicians face a significant challenge in diagnosing, treating, and discovering new treatments for SLE due to its diverse genetic and phenotypic characteristics.^1^, ^14^, ^15^, ^16^ Hence, it was crucial to recognize the risk elements associated with the advancement of SLE. Prompt identification and intervention of these factors could potentially postpone the onset of SLE. Numerous autoimmune disorders, like rheumatoid arthritis (RA), Sjögren's syndrome, systemic sclerosis, and SLE, exhibit evidence of oxidative stress, characterized by the excessive generation of reactive oxygen species (ROS), and reactive nitrogen species (RNS). Next, proteins undergo oxidative posttranslational modifications. Protein alterations can occasionally reveal neoepitopes that the immune system identifies as “foreign” and subsequently lead to the production of autoantibodies.^17^ The progression of SLE entails a rise in oxidative stress, which leads to inflammation and damage to cells. Various markers induce oxidative modifications of proteins, lipids, and DNA, leading to immune system dysfunction and a fierce autoimmune assault. Some of the indicators are improved NETosis, stimulation of the mTOR pathway, and disrupted T‐cell differentiation caused by molecular mechanisms.^18^, ^19^

According to certain scientists, bilirubin is a powerful antioxidant that can potentially offer cellular defense against detrimental stimuli.^20^ Several studies have indicated a strong correlation between serum bilirubin levels and SLE. Nevertheless, the relevance of their discoveries concerning this correlation might be considerably limited because of factors like limited sample sizes, variations in approaches, and exclusive center environments. To explore the relationship between SLE and bilirubin, we conducted this meta‐analysis by combining previous studies because no meta‐analysis had specifically examined this association. The current meta‐analysis revealed that the individuals with SLE exhibited a notable reductions in levels of serum bilirubin when compared to the healthy controls. Furthermore, there was an observed inverse correlation between serum bilirubin levels and disease activity in individuals diagnosed with SLE. In accordance with these findings, the levels of serum bilirubin were noticeably reduced in individuals with SLE who had lupus nephritis compared to those who did not have lupus nephritis.

According to reports, hyperbilirubinemia has been identified as a worrisome indication of liver malfunction, whereas slightly elevated bilirubin levels may offer protection against various diseases linked to heightened oxidative stress, by suppressing the immune system and inhibiting protein phosphorylation.^21^ Bilirubin is a byproduct of hemoglobin released by red blood cells. Generally, hemeoxygenase‐1(HO‐1) has the ability to break down heme and generate carbon monoxide, iron, and biliverdin (BV).^22^, ^23^ Subsequently, biliverdin reductase converts biliverdin to bilirubin. In the presence of hydrogen peroxide or organic hydroperoxides, bilirubin has the ability to efficiently scavenge singlet oxygen, react with superoxide anions and peroxyl radicals, and act as a reducing substrate for peroxidases.^24^ It was reported that bilirubin was found to have antioxidant and cytoprotective functions that complemented glutathione in a physiological manner.^25^ Bilirubin had been indicated to prevent the development and progress of many cardiorenal and metabolic diseases. The presence of bilirubin disrupted the interaction between vascular cell adhesion molecule 1 (VCAM‐1) and intercellular adhesion molecule 1 (ICAM‐1), thereby controlling the development of atherosclerotic lesions and inflammation in blood vessels. Additionally, it influenced the activity of T helper type 17 (Th17) cells, deactivated the NLRP3 inflammasome, and hindered the Toll‐like receptor 4/nuclear factor kappaB signaling pathway.^23^ Besides, bilirubin may protect against the deleterious effects of inflammations. SLE is acknowledged as a condition in which autoantibodies are produced and attach to self‐antigens, causing activation of the complement system and subsequently causing inflammation and harm to tissues. In SLE, the diagnostic markers for monitoring disease activity often included the reduced levels of complement proteins (C3, C4).^26^ Cecilia have demonstrated that bilirubin could inhibit the complement haemolytic cascade in sheep erythrocytes sensitized with a rabbit antisheep erythrocyte antibody. The nature of bilirubin in anticomplement might help improve complement‐mediated harm in conditions like SLE, in which antibody‐dependent complement‐mediated cell injury is involved.^27^ Typically, systemic lupus erythematosus is more common in females who are in their childbearing years. Nevertheless, perhaps because of the size of the sample, this present investigation indicated no disparity in bilirubin levels among SLE patients of both genders.

It is important to carefully consider various restrictions associated with our meta‐analysis. The data examined in this current meta‐analysis were acquired from case–control studies with limited sample sizes, and exclusive center environments. Furthermore, our meta‐analysis included variations in clinical complications, SLEDA scores for disease activity, duration of the disease, and treatments, which could potentially affect the credibility of our findings. Hence, additional studies of superior quality are necessary to authenticate the correlation between bilirubin and SLE.

## 5 CONCLUSION

This meta‐analysis revealed that serum bilirubin were significantly elevated in individuals without SLE compared to SLE patients, particularly in those with inactive SLE and without lupus nephritis. However, there were no difference between SLE patients of different sexes. Serum bilirubin concentration may serve as an indicator to assess the advancement of SLE and indicate renal complications in individuals with SLE. Nevertheless, future confirmation of these findings necessitates additional studies with larger sample sizes and multiple centers.

## AUTHOR CONTRIBUTIONS

*Conception and desig*n: Zheng Jiang and Weihua Wu. *Collection and assembly of data*: Yanxia Yu, Qiaoyu Wang, and Zheng Jiang. *Data analysis and interpretation*: Zheng Jiang and Dongmei Zhang. *Manuscript writing*: Yanxia Yu, Dongmei Zhang, Qiaoyu Wang, Weihua Wu, and Zheng Jiang. All authors read and approved the final manuscript.

## CONFLICT OF INTEREST STATEMENT

The authors declare no conflict of interest.

## Data Availability

The data sets generated and/or analyzed during the current study are available from the corresponding author on reasonable request.
